# LCMV Glycosylation Modulates Viral Fitness and Cell Tropism

**DOI:** 10.1371/journal.pone.0053273

**Published:** 2013-01-07

**Authors:** Cyrille J. Bonhomme, Kristeene A. Knopp, Lydia H. Bederka, Megan M. Angelini, Michael J. Buchmeier

**Affiliations:** 1 Department of Molecular Biology and Biochemistry, University of California Irvine, Irvine, California, United States of America; 2 Departments of Molecular Biology and Biochemistry and Division of Infectious Disease, Department of Medicine, University of California Irvine, Irvine, California, United States of America; University of Utah School of Medicine, United States of America

## Abstract

The glycoprotein (GP) of arenaviruses is glycosylated at 11 conserved N-glycosylation sites. We constructed recombinant lymphocytic choriomeningitis virus (rLCMV) featuring either additions or deletions of these N-glycans to investigate their role in the viral life cycle. N-glycosylation at two sites, T87 and S97, were found to be necessary to rescue rLCMV. Three of nine successfully rescued mutants, S116A, T234A, and S373A, under selective pressures in either epithelial, neuronal, or macrophage cells reverted to WT sequence. Of the seven stable N-glycan deletion mutants, five of these led to altered viral fitness and cell tropism, assessed as growth in either mouse primary cortical neurons or bone marrow derived macrophages. These results demonstrate that the deletion of N-glycans in LCMV GP may confer an advantage to the virus for infection of neurons but a disadvantage in macrophages.

## Introduction

The *Arenaviridae* are enveloped bi-segmented single stranded RNA viruses, with 25 species currently being recognized [1], [2], [3], [4]. Specific rodents are the principal hosts of the arenaviruses. Humans usually become infected through contact with infected rodents or via inhalation of infectious rodent excreta. Lassa, Junín, Machupo, Guanarito, and Sabia viruses are known to cause hemorrhagic fever in West Africa, Argentina, Bolivia, Venezuela, and Brazil, respectively [2], [5], [6], [7], [8], [9], [10], [11], [12].

The prototypic arenavirus, lymphocytic choriomeningitis virus (LCMV), is an agent of acute central nervous system disease as well as a cause of congenital malformations [13], [14], and has been associated on several occasions with fatal outcome in organ-transplanted recipients [15], [16], [17] and immune compromised patients [18], [19]. The high degree of genetic variation among geographic and temporal isolates of the same arenavirus species supports the concept of continued emergence of new pathogens [20], as recently proven by the identification two novel arenaviruses: Lujo and Dandenong; isolated respectively from a fatal outbreak [3], [21] and from fatal organ transplants [22].

LCMV virions are composed of a nucleocapsid surrounded by a lipid bilayer that presents spikes of glycoproteins (GP) [23]. The initial step in LCMV infection involves interaction of GP with the cellular receptor on target cells [24]. After internalization of the virions within vesicles, LCMV GP mediates fusion of the viral and cellular membranes, resulting in delivery of the nucleocapsid into the cytoplasm [25], [26], [27], [28], [29].

**Figure 1:**
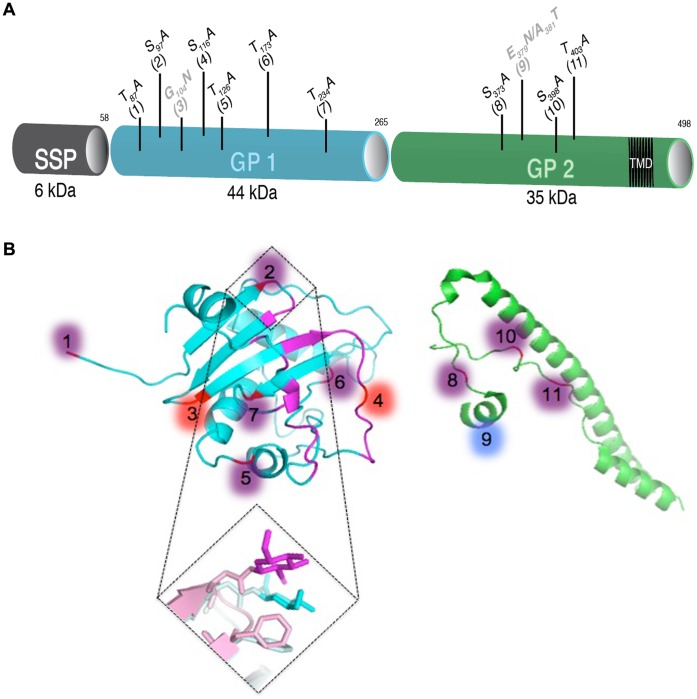
LCM virus glycoprotein overview. (A) Schematic view of the glycoprotein and localization of the N-glycosylation site mutations. The signal peptide SSP in grey (aa 1–58), the precursor glycoprotein GPC (aa 59–498), the subunits GP1 in blue (aa 59–265) and GP2 in green (aa266–498) containing the transmembrane anchor (aa 439–456, black) are shown. The eleven conserved N-glycosylation sites across arenaviruses are represented. Mutations leading to: (i) deletion of N-glycan on LCM virus in black, (ii) addition of N-glycan on LCM virus in grey. (B) Modified crystal structure of Machupo virus GP1. Residues in direct interaction with human transferrin receptor 1 are shown in magenta. Positions of N-linked glycans conserved by both LCMV and Machupo virus are purple. Those conserved only by LCMV are red and those conserved only by Machupo are blue. (C) Crystal structure of LCMV GP2 ectodomain. Positions of the glycosylated asparagines from consensus sequence are shown in red (8–11). (D) Crystal of Machupo virus GP1 (Light cyan) superimposed on receptor bound Machupo GP1 (Light pink) with glycan 2 shown in cyan and magenta, respectively.

The 498-amino-acid glycoprotein complex (GPC) of LCMV consists of three domains (Figure 1A). The stable signal peptide SSP (GPC residues 1 to 58) is co-translationally cleaved by signal peptidase. The precursor GPC is cleaved into GP1 (residues 59 to 265) and GP2 (residues 266 to 498) by the cellular protease SK-1/S1P [30] and forms a spike complex [31]. GP1 of arenaviruses contain between 4 and 11 predicted N-glycosylation sites. GP1 contains the receptor-binding site, antibody neutralization sites, and is non-covalently associated with GP2 [32], [33]. GP2 contains 3 to 4 highly conserved N-glycosylation sites and a transmembrane region that anchors the GP complex in both the lipid bilayer of the cell membrane and the viral envelope [32], [34].

N-linked glycosylations are important for both protein folding and function [35], [36]. Wright *et al*. demonstrated that N-linked glycosylations play a role in masking neutralizing epitopes for LCMV [37]. Epitope GP-1D is one such conformational epitope and is dependent on the presence of N-linked glycosylation [37]. This has also been shown for other viruses, including influenza C [38] and human immunodeficiency virus [39]. We have previously confirmed the role of glycans in neutralizing epitope masking as well as demonstrated that LCMV GP N-glycosylation selectively affects a variety of downstream GP functions, including expression, cleavage, pH-dependent fusion, and formation of infectious particles [40]. The emergence of reverse genetic tools allowed us to engineer recombinant LCMV (rLCMV) from cDNA [41]. This allowed for the possibility of shuttling our previously described N-glycosylation mutants directly into recombinant viruses to further study the role of the conserved N-glycosylations in the viral life cycle. This brings us one step closer to evaluating rLCMV as a vaccine candidate for arenavirus diseases. In the present study, we rescued rLCMV N-glycosylation mutants and assessed the roles of each single N-glycan in viral fusion, overall viral fitness, and cell tropism.

## Materials and Methods

### Cell Lines and Primary Cells

DBT murine astrocytoma [42], BHK-21 hamster kidney fibroblast (ATCC CCL-10), L929 murine fibroblast (ATCC CRL-2148), HEK 293T human embryonic kidney epithelial (ATCC CRL-11268), Vero E6 African green monkey kidney epithelial (ATCC CRL-1586), OBL21A mouse olfactory bulb neuronal, and RAW 264.7 mouse macrophage (ATCC TIB-71) cells lines were maintained in Dulbecco’s modified Eagle’s medium (DMEM) supplemented with 10% fetal bovine serum (FBS) and 1% penicillin-streptomycin. Mouse cortical neurons were isolated from C57BL/6 mice as previously described [43] and cultured for 4 days before infection assays. Mouse bone marrow-derived macrophages (BMDM) also from C57BL/6 mice were generated as previously described [44] and cultured in DMEM-10% medium supplemented with 20% L929 conditioned media for 6 days before infection assays.

### N-glycosylation Mutation Design

To ensure tracking and genetic stability, a triplet of silent mutations was included every 500 bp along the glycoprotein sequence for use as genetic markers. Desired mutations in the N-glycosylation sites were inserted in the glycoprotein cDNA. Mutations were designed to either modify the N-glycosylation sites already present (deletion of N-glycosylation) or to create a hyper-glycosylated (addition of N-glycosylation) LCMV glycoprotein. These mutations where designed as follows: a triplet mutation was inserted at each glycosylation site in order to replace the S/T with an A or to create a new glycosylation site. These triplets contained at least two transversions (A ↔ T or A ↔ C or G ↔ C or G ↔T) to create a high genetic barrier against reversion.

### Virus Strains, Infection, and Titration

Recombinant viruses were made using reverse genetic technology described previously [45], [46]. Virus stocks were generated by serial passage in BHK-21 cells and sequenced to ensure that no major genetic changes had occurred during passage of the viruses. The LCMV-Armstrong clone 4 (Arm-4), clone 5 (Arm-5) and cl-13 were used throughout these studies [37]. Cells were infected at MOI 0.01 or MOI 2 for 1 h adsorption at 37°C 5% CO_2_ under rocking conditions followed by two washes with PBS and addition of fresh media. MOI was determined as FFU/cell and virus titer as FFU/ml. Cells and collected supernatants were flash frozen then subjected to 2 cycles of freeze/thaw at various times T1, 4, 7, 19, 24 and 48 h post infection. The resulting total virus titers were assessed by immunofluorescence on Vero E6 cells and expressed as FFU/ml as described previously [47].

### Cell Fusion Assay

DBT cells were infected at MOI 5 for 2 h then were exposed to pH 5 buffered medium for 1 h then returned to neutral pH for 1 h. Cells were fixed with 4% PFA and stained with Giemsa. The extent of syncytium formation was quantified using the module, object count, from Nikon NIS software for 10 random images at 100X magnification. Percentage of cell fused was normalized to WT. Statistics were analyzed by one-sample t test P<0.0001 compared to WT.

### Modification of Crystal Structure

Crystal structures for Machupo GP1 (2WFO) [48], Machupo GP1 bound to the transferrin receptor (3KAS) [49] and LCMV GP2 (3MKO) [50] were obtained from PDB and rendered using the PyMOL Molecular Graphics System, Version 1.3 Schrödinger, LLC.

### Statistical Analysis

All statistical analyses were generated using Prism 4 (GraphPad) software using one-sample t test P<0.0001 compared to WT. Virus production calculated as follows: Log 2 (rLCMV mutant mean titer at 24 h post infection/rLCMV WT mean titer at 24 h post infection) (N = 4).

## Results

### Rescue of rLCMV with Deletion or Addition of N-glycan on the Glycoprotein

Eleven conserved sites (≤50% conservation) that allow the attachment of N-linked oligosaccharides are predicted for arenavirus GP (Table 1). We previously demonstrated that these sites were indeed glycosylated *in vitro* on the LCMV glycoprotein with one exception [40]. Using reverse genetics, we engineered rLCMV with deletion or addition of N-glycans on the WT LCMV glycoprotein [41]. To ensure tracking and genetic stability, a triplet of silent mutations was included every 500 bp along the glycoprotein sequence for use as genetic markers. The rLCMV carrying these markers exhibited no differences as compared to the parental LCMV Arm 5 strain. Desired mutations in the N-glycosylation sites were inserted in the glycoprotein cDNA. Mutations were designed to either modify the N-glycosylation sites already present (deletion of N-glycosylation) or to create a hyper-glycosylated (addition of N-glycosylation) LCMV glycoprotein (Figure1A & Table 1).

**Table 1:** Glycosylation site conservation and recombinant LCMV genetic stability.

Mutation	Conservation^a^		Stability^b^		
	OW	NW	Epithelial	Neurons	Macrophages
WT Arm 5	n/a	n/a	10	10	10
F260L	n/a	n/a	10	10	10
*Deletion of N-glycosylation site*					
T87A (1)	8/8	15/20	0	0	0
S97A (2)	8/8	20/20	0	0	0
S116A (4)	7/8	7/20	1	0	0
T126A (5)	8/8	18/20	10	10	10
T173A (6)	7/8	20/20	10	10	10
T234A (7)	8/8	17/20	10	10	6
S373A (8)	8/8	20/20	7	3	3
S398A (10)	8/8	19/20	10	10	10
T403A (11)	8/8	20/20	10	10	10
*Addition of N-glycosylation site*					
G104N (3)	5/8	9/20	10	10	10
E379N/A381T(9)	5/8	20/20	10	10	10

Specific mutations of N-glycosylation sites were engineered to delete or add N-glycan on the LCM virus glycoprotein.

a Conservation of the glycosylation site from alignment of arenavirus GP sequences performed using CLC Sequence Viewer software. The 8 Old Word arenaviruses are: Lassa virus (LASV), X52400; Ippy virus (IPPYV), DQ328877; Mobala virus (MOBV), AY342390; Mopeia virus (MOPV), DQ328874; Morogoro virus (MORV), EU914103; Dandenong virus (DANV), EU136038; Lymphocytic choriomeningitis virus (LCMV), AY847350; Lujo virus (LUJV), FJ952384; while the 20 New World arenaviruses are: Pirital virus (PIRV), AF277659; Allpahuayo virus (ALLV), AY012687; Pichinde virus (PICV), NC_006447, Parana virus (PARV), AF512829; Flexal virus (FLEV), AF512831; Oliveros virus (OLVV), U34248; Latino virus (LATV), AF512830; Machupo virus (MACV), NC_005078; Junin virus (JUNV), D10072; Tacaribe virus (TCRV), NC_004293; Chapare virus (CHAV), EU260463; Sabia virus (SABV), NC_006317; Guanarito virus (GTOV), NC_005077; Amapari virus (AMAV), AF512834; Cupixi virus (CPXV), AF512832; Skinner Tank virus (SKTV), EU123328; Whitewater Arroyo virus (WWAV), AF228063; Catarina virus (CATV), DQ865245; Tamiami virus (TAMV), AF512828; Bear Canyon virus (BCNV), AF512833.

b Stability controlled by sequencing of GP for each rescued virus mutant 72 h post infection in (i) epithelial cell lines: Vero E6 and HEK293T, (ii) neurons: cell line OBL21A and primary mouse cortical neuron, (iii) macrophages: cell line RAW264.7 and bone marrow derived macrophages. Stability was measured on a scale from 0–10 and represents the stability of the mutants during 10 passages, on epithelial cells, or during a 72 h infection, on primary cells. ‘0′ means no rLCMV rescued and ‘10′ mutation stable for more than 10 passages on epithelial cells and more than 72 h on primary cells.

Nine of the eleven N-glycosylation mutants were rescued. The deletion of the first two N-glycosylation sites on GP1 (T87A #1 and S97A #2) failed to generate recombinant virus. We subsequently investigated the stability of the rescued rLCMV mutants during ten virus passages on an epithelial cell line and during 72 h infection in neuron cell lines, macrophage cell lines, mouse primary cortical neurons, and mouse bone marrow derived macrophages (BMDM) (Table 1). Three rescued rLCMV mutants (S116A #4, T234A #7, and S373A #8) appeared unstable to varying degrees. The most critical mutation, S116A, reverted to the WT sequence within 24 h post-infection, rendering purification and viral expansion impossible. The second mutation, T234A, reverted to WT sequence only in macrophages, both in the cell line and in primary cells, between 24–48 h post-infection. However, T234A remained stable for up to ten passages in epithelial and neuron cell lines. The last unstable mutation, S373A, was stable for several passages, though mutation was observed after seven passages in epithelial cells and within 24 h in neurons and macrophages, both in the cell lines and in primary cells. This mutation did not revert to WT sequence (serine) but instead featured a compensatory mutation replacing the alanine with a threonine. This compensatory mutation restored a functional site due to the redundancy of the glycosylation motif N-X-S/T. (Table 1).

### Hyper-glycosylation of N-glycan in GP1 of LCMV (G104N #3 mutation) Reduces Virus Fusion

**Figure 2:**
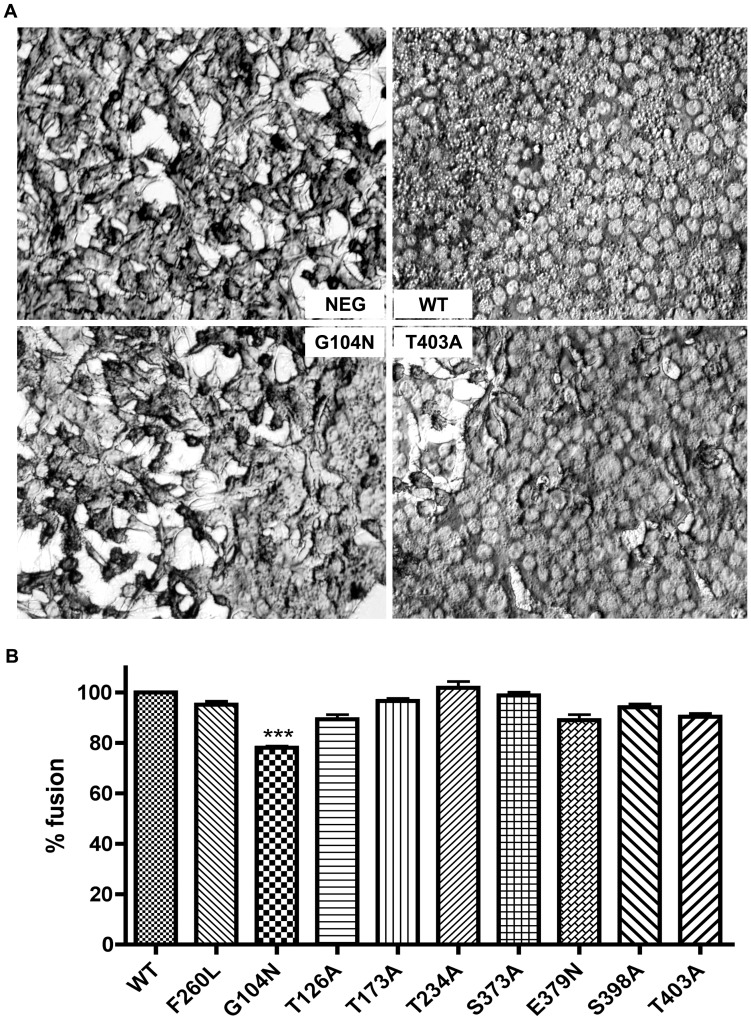
Addition of N-glycan in GP1 of LCM virus causes reduction in virus fusion. (A) DBT cells were infected at MOI 5 for 2 h then exposed to media pH 5 triggering fusion mediated by the glycoprotein. (B) Percentage of cells fused normalized to WT measured by microscopy using module object count from Nikon NIS software N = 10. Statistics analyzed by one-sample t test P<0.0001 compared to WT.

To investigate if the addition or deletion of a single N-glycan on the LCMV glycoprotein affected viral fusion, we tested each rLCMV in a cell fusion assay. DBT cells were infected with the rLCMV mutants and their ability to promote syncytium formation at pH 5 was assessed by microscopy. A representative example of the fusion assay is shown in Figure 2A. Only the G104N #3 mutation, which leads to the hyper-glycosylation of GP1, significantly reduced the percentage of fusion as normalized to WT. However, rLCMV G104N was still able to promote 78% of fusion compared to WT. All other mutant rLCMV exhibited fusion efficiencies similar to the WT (Figure 2).

### rLCMV N-glycan Mutants Exhibit Differing Fitness in Neurons *vs.* Macrophages

To assess whether the addition or removal of these N-glycans changes the overall fitness of the virus, we performed infections on Vero E6 cells using the various N-glycan mutant viruses at an MOI of 0.01 and looked for total viral production at 24 hour increments over a total period of 72 hours. All the rLCMV examined were able to infect and produce virus in similar amounts as compared to WT. Since no significant differences were observed in Vero E6 cells, we tested HEK293T and BHK-21 cells with similar findings. This suggests that these N-glycans had little or no effect on virus fitness in epithelial cell lines (data not shown).

**Figure 3:**
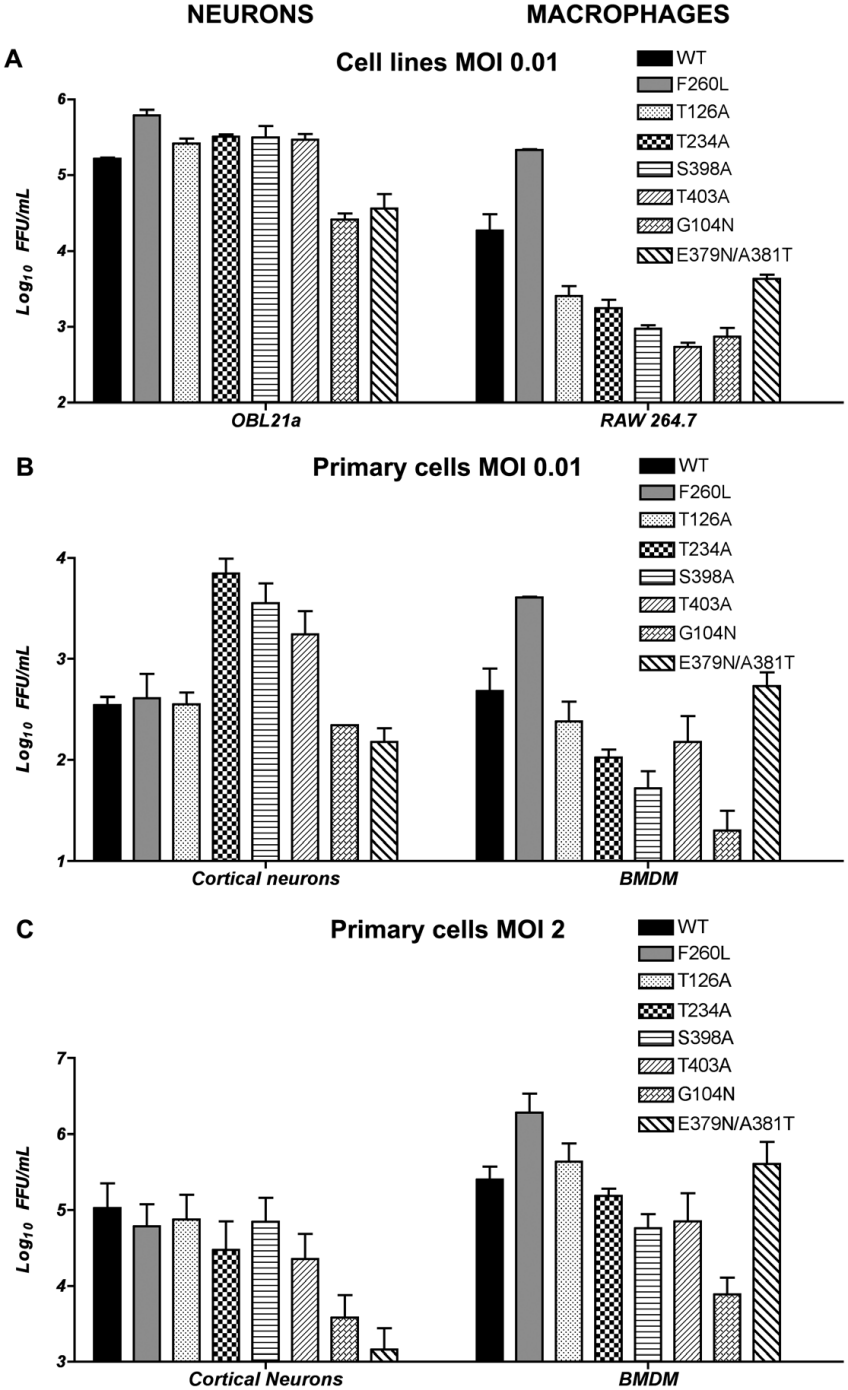
Neurons *vs.* macrophages: rLCMV N-glycan mutants production comparison. Titer of rLCMV N-glycan mutants measured 24 h post infection. (A) In mouse neuronal cell line OBL21a *vs.* macrophage cell line RAW 264.7 at MOI 0.01. (B) In mouse primary cortical neurons *vs.* primary BMDM MOI 0.01 and (C) same primary cells but MOI 2. Virus titer was measured by immune focus assay at T24 h post infection (N = 4).

The selection of organ-specific viral variants known to occur during WT viral infection led us to examine the effect of these N-glycan mutant viruses in different cell types [20]. The prototypic member of the immunosuppressive variant of LCMV Arm, cl-13, predominates in lymphocytes and macrophages [51], [52], whereas the LCMV Arm parental strain predominates in the central nervous system (CNS) and outcompetes cl-13 virus in a cultured neuronal cell line [53]. We tested our N-glycan mutant rLCMV in OBL21a neuron *vs.* Raw 264.7 macrophage cell lines at low MOI (0.01) then confirmed our results in primary mouse cortical immature neurons *vs.* BMDM at low MOI (0.01) and high MOI (2) (Figure 3 & Table 2). Differences in virus N-glycan mutant fitness in neurons *vs.* macrophages were already significant in cell lines and were increased in primary cells. Two N-glycan deletions, T126A #5 and T173A #6, exhibited similar growth curves compared to WT, suggesting that these two N-glycans are not required for virus infectivity and survival in either neurons or macrophages. We included as a control in these studies a mutant rLCMV featuring the GP mutation responsible for the phenotype of the cl-13 immunosuppressive variant: F260L [54]. As expected, the F260L showed increased virus titer at 24 h post infection in both primary and continuous mouse macrophage cell lines, and was independent of the multiplicity of infection. The titers of the F260L mutant virus did not differ from the parental WT in immature primary cortical neurons.

**Table 2:** Deletion or addition of N-glycan on the glycoprotein modifies LCM virus fitness towards mouse neurons or macrophages.

Mutation	Cell line MOI 0.01		Primary cell MOI 0.01		Primary cell MOI 2	
	*OBL21a*	*RAW 264.7*	*Neurons*	*BMDM*	*Neurons*	*BMDM*
F260L	**+1.91 ↑**	**+5.92 ↑**	+0.22	**+3.85 ↑**	+0.58	**+2.37 ↑**
*Deletion of N-glycosylation site*						
T126A (5)	+0.67	−0.46	+0.02	−0.22	−0.58	−0.67
T234A (7)	+0.96	−1.00	**+4.32 ↑**	−1.41 ↓	−1.82 ↓	−1.83 ↓
S398A (10)	+0.94	−1.90 ↓	**+3.34 ↑**	−1.41 ↓	−0.57	−3.32 ↓
T403A (11)	+0.84	−2.69 ↓	**+2.32 ↑**	−0.9	−2.21 ↓	−4.45 ↓
*Addition of N-glycosylation site*						
G104N (3)	−2.66 ↓	−2.24 ↓	−0.66	−3.81 ↓	−4.78 ↓	−6.69 ↓
E379N/A381T (9)	−2.18 ↓	−1.22 ↓	−1.22 ↓	+0.94	−6.20 ↓	+1.00

Virus production at 24 h post infection for the different rLCMV glycosylation mutants compared to WT in mouse cell line or primary cell and at low (0.01) or high (2) MOI. Virus production calculated as follows: Log 2 (rLCMV mutant titer at 24 h post infection/rLCMV WT titer at 24 h post infection) (N = 4). Decreased virus production −1 (grey ↓) ≤ wt virus production ≥1 increased virus production (**bold ↑**).

The three N-glycosylation deletion mutants (T234A #7, S398A #10, and T403A #11) all exhibited similar fitness. These three mutants all exhibited decreased fitness in macrophages, whether in cell lines or primary cells and at either low or high MOI, but were still able to persist in these cells. In neuronal cell lines and primary cells, these deletion mutants demonstrate an increased fitness at low MOI (0.01). However the fitness was decreased at high MOI (2) in primary cortical neurons. (Figure 3, 4 & Table 2).

**Figure 4:**
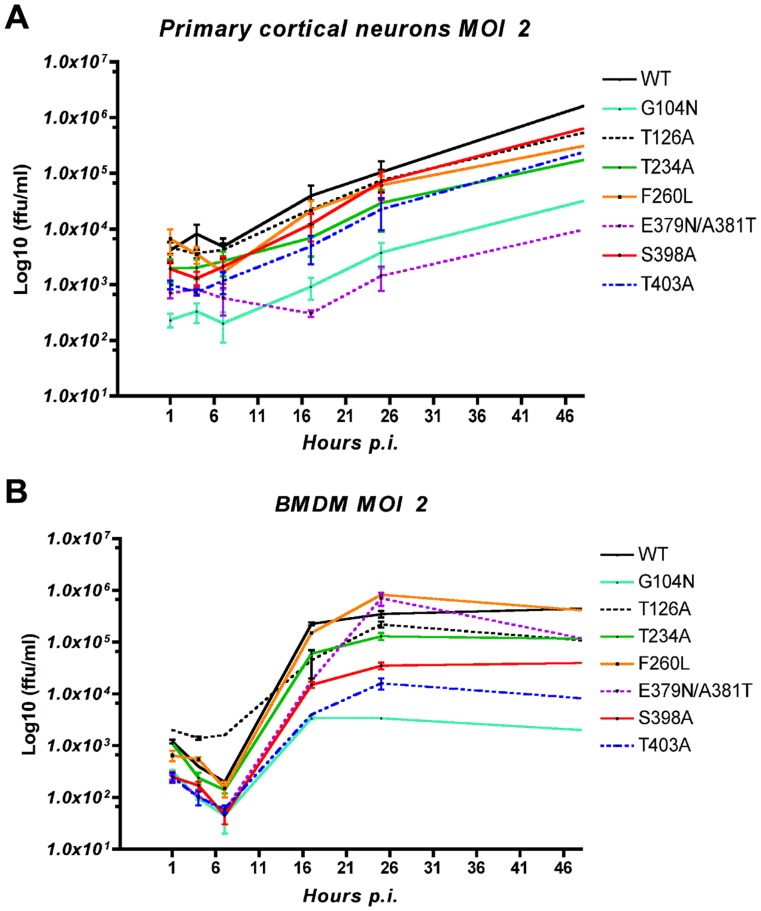
Mouse primary cortical neurons *vs.* BMDM: rLCMV N-glycan mutants growth curve. Infection of (A) mouse primary cortical neurons and (B) BMDM were performed at MOI 2. Total virus titer was measured by immune focus assay from cells and supernatant at T1, 4, 7, 17, 24 and 48 h post infection (N = 4).

The hyper-glycosylation mutants, either in GP1 (G104N #3) or GP2 (E379N/A381T #9), produced rLCMV with significantly decreased viral fitness in neurons. Addition of N-glycan on G104N negatively affects the mutant’s fitness in both macrophages and neurons, either in cell lines or primary cells at either low or high MOI. Specifically, G104N showed a decrease in virus production in primary cell macrophages of −3.81 at low MOI and −6.69 at high MOI (Table 2). Conversely, the other addition of glycan in GP2, E379N/A381T #9, exhibited a fitness toward macrophages that was the same as the WT strain, despite its lower fitness in neurons.

### rLCMV N-glycan Mutants Exhibit Differing Growth in Neurons *vs.* Macrophages

To further investigate the affinity of the deglycosylated mutants (T234A #7, S398A #10 and T403A #11) and the hyper-glycosylated mutants (G104N #3 and E379N/A381T #9) towards either neurons or macrophages we chose to assess virus growth in primary cells at high MOI by measuring total virus titer at 1, 4, 7, 17, 24, and 48 h post infection (Figure 4.). The first striking difference is the type of growth curves obtained. While we observed almost linear growth in mouse primary neurons, BMDM infection exhibited a one step growth curve with an early decrease in virus titer followed by an exponential growth between 6 and 16 h post infection with a Log_10_ Slope = Log_10_(ΔTiter/ΔTime) >2.5, reaching a plateau at 24 h. The decreased fitness of the two hyper-glycosylated rLCMV mutants is explained by a lower total virus titer at T1. Total virus titer at T1 was measured after 1 h adsorption at 37°C followed by two washes with PBS and addition of fresh media. The cells and fresh media were then immediately frozen and thawed twice to release virus. Therefore, the T1 titer represents the number of infectious viruses still bound to the cell or those in early endocytosis prior to fusion and entry. Since all virus stocks and dilutions were carefully quantified, we can conclude that this lower virus titer at T1 is the result of a deficiency in the early infection processes. The hyper-glycosylation mutant localized on GP2, E379N/A381T, exhibited a unique feature during primary neuron infection, with an initial drop in virus titer up to the 16 h time point followed by constant linear growth through later time points. Three deglycosylation mutants, T234A, S398A, and T403A, however, do not exhibit a significantly different growth curve compared to WT LCMV in primary neurons.

BMDM growth curves give us more information as to why these three rLCMV deglycosylation mutants have a lower fitness in macrophages. First, both S398A #10 and T403A #11 have decreased virus titer at T1 post infection in BMDM but not in neurons, suggesting a specific defect in early steps of infection in BMDM. Second, T234A #7 as well as S398A #10 and T403A #11 all have a decreased exponential growth with Log_10_Slope of 3.7, 3.1, and 2.6 respectively compared to the WT Log_10_ Slope of 4.3. This leads to a lower virus titer for the mutants upon reaching the plateau, suggesting a negative effect of the deglycosylation on virus replication and/or spread. While both of the rLCMV hyper-glycosylation mutants (G104N #3 and E379/A381T #9) exhibited decreased T1 titers, their growth curves during later time points behaved differently. G104N #3 presented a shorter and decreased exponential growth (Log_10_Slope  = 2.5) and plateau 8 h earlier than the WT. Interestingly, mutant E379N/A381T #9, despite having a lower titer at T1, had 8 more hours of exponential growth than the WT, leading to a WT-like titer in macrophages at 18 h post adsoption.

## Discussion

This study, together with our previous *in vitro* study showing how N-glycosylation modulates glycoprotein expression and function [40], provides a deeper understanding into the role of N-linked glycosylation of GPC in LCMV infection. Here we used rLCMV carrying N-glycosylation mutations in the context of a complete viral infection to explore how the different conserved arenavirus N-glycans affect LCMV. Eleven glycosylation sites (N-X-S/T) are more than 50% conserved in both Old and New World arenaviruses. LCMV Arm-5 glycoprotein possesses nine of these N-glycosylation sites: six sites on GP1 and three sites on GP2 (Figure 1A). We chose to examine the two missing conserved N-glycosylation sites in LCMV since this is the prototypic arenavirus and because LCMV can be handled in a biosafety level two setting. Mutations were designed by reverse genetics to either remove the N-glycosylation sites already present or create the missing conserved sites (based on conserved N-glycosylation sites from Lassa virus strain Nigeria) on the LCMV glycoprotein (Figure1A & Table 1). In the present study, we clearly demonstrate that GP associated N-linked glycosylation sites do not contribute equally to virus synthesis, entry, and cell tropism.

Although the first two-thirds of GP1 is the most variable among arenaviruses, the first two N-glycosylation sites are heavily conserved (82% and 100% respectively). Removal of these N-glycans makes the rescue of viable rLCMV impossible. This is in accordance with our previous *in vitro* findings demonstrating that transfected LCMV glycoprotein mutated at these positions had poor expression levels and failed to be cleaved or to mediate fusion and infection.

Bowden, *et al*. have shown that deglycosylation of Machupo GP1 results in precipitation of the protein. This suggests that, as reported in other systems, arenavirus glycans serve to render an otherwise hydrophobic molecule hydrophilic and soluble [55]. This role of protein solubilization is supported by our previous findings [40] and would explain why the first N-glycan of GP1 is critical for a functional glycoprotein. As demonstrated by the Machupo GP1 crystal structure (Figure 1 B and D), where the first glycosylation site is not included, Bowden, *et al*. suggest the pyranose ring of the second GlcNAc forms a stacking interaction with Phe98 while the rest of the glycan covers other solvent exposed aromatic residues. This Phe98 was later shown to interact with the human transferrin receptor 1 (hTfR1) [49]. Although N-linked glycans are not involved in receptor binding, according to Abraham J. *et al*., the interaction between the glycan and the amino acid at position 98 seems to be present when the glycoprotein is not bound to its receptor (Figure 1D). Therefore, we propose that the second N-glycan on GP1, in addition to solubilizing the glycoprotein, is able to mask key residues responsible for receptor binding and is flipped away when the glycoprotein is bound to its receptor. This hypothesis could explain why it has been difficult to produce neutralizing antibodies against the GP1 receptor-binding domain [56]. In LCMV this second glycan may interact with and block solvent exposure of aromatic or non-polar residues involved in α-dystroglycan binding.

The fourth site of N-glycosylation (S116 #4), though the least conserved among New World arenaviruses, is critical for LCMV. Our previous analysis of this deglycosylated mutant glycoprotein in transfected cells suggests this N-glycan is expendable for glycoprotein solubilization. However, while it is possible to rescue rLCMV lacking this fourth N-glycosylation site, the virus reverts within a single generation to the WT sequence (Figure 1A & Table 1). This, in conjunction with our previous pseudotyping experiments, signifies a selective pressure to keep an N-linked glycan at this position. This glycan may be required to play another essential role beyond solubility and virion formation, considering the crystal structure of Machupo GP1 provides a possible role for this glycosylation in LCMV [49]. Assuming LCMV and Machupo GP1 share structural homology, the surface region for binding their respective receptors may be similar. If this were the case for LCMV, this glycan would sit in a loop that may directly interact with α-dystroglycan (Figure 1B). This could explain the essential role and selection pressure of this N-glycan for the arenaviruses.

The first N-glycan mutant in GP2, S373 #8, while stable in epithelial cells for several passages, was subjected to pressure resulting in rapid reversion, restoring the N-glycosylation site when infecting primary neurons or macrophages. This reversion resulted in a single genetic transition (G ↔ A) and restored a threonine instead of a serine. This compensatory mutation restored a functional site due to the redundancy of the glycosylation motif N-X-S/T. It is likely the reversion did not restore a WT serine because this would have required two mutation events, one transition and one transversion, creating a much higher genetic barrier. This suggests a cell-type specific selective pressure for conservation of the first N-glycan on GP2 to retain the ability to infect primary neurons or macrophages.

rLCMV missing the last N-glycan in GP1 T234 #7 exhibited an even more specific cell type pressure. The rLCMV mutant T234A was very stable in epithelial and neuron cell lines as well as in primary cells. Infection of either macrophage cell lines or primary BMDM cells resulted in reversion by a single genetic transition (G ↔ A) to the WT protein sequence between 24 h and 48 h post infection, suggesting a macrophage-specific pressure to keep this N-glycan. The reversion appears relatively late during the infection, explaining why the rLCMV T234A mutant produced significantly less virus during the first 24 h of the infection. Despite 100% reversion to WT sequence, the virus never recovered and barely survived past 48 h when infecting BMDM at low MOI. The rLCMV virus was eliminated from infection at MOI = 0.01 in mouse BMDM from one out of four assays and did not yield more than one hundred infectious particles per milliliter in the remaining three assays. This suggests the reversion to WT appears too late during infection and the low number of surviving viruses are insufficient (around 10^2^ FFU/mL) to survive the BMDM antiviral response.

At a high MOI in BMDM, the reversion to WT appears between 18 h and 24 h. This explains why we observed a WT like growth curve with less virus production (Figure 4.). A higher MOI increases the speed at which T234A rLCMV reverts to WT, suggesting a competition between WT and T234A; a sign of either an undetectable WT subpopulation already present in the inoculum or the occurrence of a bottleneck. This behavior is characteristic of defective LCMV, where increased MOI of T234A rLCMV increases the production of defective virus competing with the normal T234A rLCMV [57]. This explains why an increased inoculum resulted in a less productive infection in neurons. Having a N-glycan on the last N-glycosylation site of GP1 is necessary for virus survival in macrophages but is optional in neurons. This demonstrates the role that a single N-glycan at the end of GP1 can play in virus fitness and cell tropism. It also provides examples of viral fitness being environment-dependent as has been shown for modulation of HIV fitness by the host environment [58] as well as for LCMV where specific environment-dependent mutations were selected in the brain or the spleen [59].

In contrast, two N-glycosylation deletions at sites T126A #5 and T173A #6 in GP1, while stable, did not seem to have any effect on virus fitness or cell tropism. This is not surprising for rLCMV mutant T173A since this mutation leads to the naturally occurring variant, LCMV Arm4, which lacks the N-glycan and fails to prevent the generation of neutralizing antibodies against epitope GP-1D [37]. However, the similarity to WT rLCMV for mutant T126A is surprising since our previous *in vitro* studies demonstrated decreased infectivity [40]. An examination of the homologous residues in the Machupo GP1 structure suggests that neither N-glycan T126A nor T173A participate directly in receptor binding due to their distance from the residues known to interact with human transferrin receptor 1 [49]. rLCMV mutant T126A displays a similar pattern to T173A encouraging the idea that N-glycan at position T126 may play a similar role of epitope masking as demonstrated for T173A while not directly impacting viral fitness.

The effect of N-glycan deletion on viral fitness in neurons and macrophages is best demonstrated by the rLCMV mutants S398A #10 and T403A #11. At low MOI, both stable mutant rLCMV lead to a similar pattern of decreased virus production in macrophages and increased virus production in neurons. At high MOI, near WT virus production suggests the same recurrent effect of defective virus competion in neurons. The deletion of these N-glycans appears to be a definite advantage in neurons but a disadvantage when it comes to macrophages. We demonstrated that this disadvantage comes from a less efficient early infection and less efficient virus replication and/or spread. The detrimental consequence of N-glycan deletion on virus infectivity has been extensively studied with HIV [60], [61], [62] and other viruses [63], [64]. However, to the best of our knowledge, no studies have looked at the effect of N-glycan deletion on virus infectivity in neurons. The fact that these N-glycosylation mutants, S398A and T403A, are only 5 amino acids apart and are located just external to the transmembrane domain of GP2 suggests a deeper involvement of the GP2 glycoprotein than just its obvious anchor function.

Hyper-glycosylation of LCMV GP also results in a change in viral fitness. The rLCMV mutants G104N #3 and E379N/A381T #9, while genetically stable, exhibited a change in tissue tropism. Addition of a glycan at position 104 on LCMV GP1 decreased the fusion efficiency of the virus. This is consistent with what has been seen for Newcastle Disease virus, where hyper-glycosylation of the homologous attachment protein blocks fusion and protein interaction with the fusion partner without changing the attachment protein’s structure [65]. The G104 mutation significantly compromises virus survival in macrophages with little to no effect in neurons, taking into account its lower fusion efficiency. These data suggest that the addition of a single N-glycan alone is able to impair virus survival, possibly through reduction of fusion efficiency. Taking into account that G104N exhibited a defect in early infection, the shorter exponential growth suggests that it is not the only factor causing the poor fitness of G104N in macrophages.

The hyper-glycosylation in GP2 E379N/A381T #9 did not impair the fusion efficiency of the virus but did result in a mild viral fitness modification that was disadvantageous in neurons but had no effect in macrophages. Curiously, this is the exact opposite effect of the N-glycan deletion rLCMV mutants. The fact that the E379N/A381T mutant compensated by having a longer exponential growth resulting in a WT-like fitness in macrophages despite exhibiting a lower titer at T1 suggests an overall increased fitness in macrophages when it comes to virus replication or spread.

In an attempt to understand how the N-glycans could affect the virus fitness and cell tropism we challenged the rLCMV for stability at 4, 25, 37 and 45°C for up to 6 h. All rLCMV exhibit the same resistance as the WT (data not shown), leading us to presume that the same amount of virus was available to infect the cells and that the differences in virus production do not result from infections with less or more viable viruses. We then examined the adsorption efficiency for all rLCMV on Vero E6 cells for 1 h by looking at the disappearance of viruses from the supernatant at 4°C. The cells adsorbed the same amount of viral particles by 15 min without any significant differences compared to WT virus adsorption (data not shown). These similarities with the WT virus are not surprising since we are looking at successfully rescued rLCMV. It appears that in order to be able to rescue a viable mutant rLCMV, it needs to be physically stable against outside stressors, attach to its target efficiently, and mediate fusion relatively efficiently.

Our studies show that, apart from N-glycans T126A #5 and T173A #6, each conserved N-linked glycan in the arenavirus glycoprotein has important effects on viral fusion, infectivity, and viral fitness. These results suggest roles for these N-glycosylation sites in both the early steps of infection and later during virus replication and/or spread. More broadly, our results may suggest that a more exposed LCMV GP (missing a glycan) is more suited to successfully infect neurons or may even give the virus some growth advantages, whereas a less exposed LCMV GP (added N-glycosylation) is definitively at a disadvantage for neuron infection. The opposite appears true when it comes to macrophage infection, where hyper-glycosylation mutant (E379N/A381T #9) is preferred and a more exposed mutant is at a disadvantage.

Further studies are needed to challenge these rLCMV N-glycosylation mutants in animal models to test if selectively deglycosylated rLCMV are good vaccine candidates. We have already demonstrated the role of N-glycan in glycoprotein expression, processing, fusion, and infectivity–this new data enhances our understanding of the key roles played by these N-glycans; not only for the virus glycoprotein functionality but for overall viral fitness in different cell types as well. This reinforces the importance of recombinant virus studies for a full understanding of mutations in the context of a complete virus and the interaction of the different viral proteins during the viral life cycle.
